# Newborn Screening for Sickle Cell Disease Among Tribal Populations in the States of Gujarat and Madhya Pradesh in India: Evaluation and Outcome Over 6 Years

**DOI:** 10.3389/fmed.2021.731884

**Published:** 2022-02-15

**Authors:** Pallavi Thaker, Roshan B. Colah, Jignisha Patel, Bhavesh Raicha, Abhishek Mistry, Vishal Mehta, Yazdi Italia, Shrey Desai, Kapilkumar Dave, Rajasubramaniam Shanmugam, Kanjaksha Ghosh, Malay B. Mukherjee

**Affiliations:** ^1^Department of Haematogenetics, Indian Council of Medical Research (ICMR)-National Institute of Immunohaematology, Mumbai, India; ^2^Valsad Raktadan Kendra, Valsad, India; ^3^Society for Education Welfare and Action (SEWA) Rural Kendra, Jhagadia, India; ^4^Department of Genetic Disorders, Indian Council of Medical Research (ICMR)-National Institute for Research in Tribal Health, Jabalpur, India

**Keywords:** newborn screening, sickle cell anemia, tribal population, comprehensive care, Gujarat, Madhya Pradesh, India

## Abstract

Sickle cell disease (SCD) poses considerable public health problems in India. This study was undertaken to understand the clinical course of SCD among children identified during newborn screening programmes in Gujarat and Madhya Pradesh where the frequency of the HbS gene is high. A total of 8,916 newborn babies 8,411 from Gujarat and 505 from Madhya Pradesh were screened over 6 years (2010–2016) using HPLC and the diagnosis was confirmed by molecular analysis in a subset. A total of 128 babies (122 Gujarat, 6 Madhya Pradesh) were identified with sickle cell disease, of whom 87 (69 HbSS, 18 HbS-β thalassemia) from Gujarat were followed for 0.5–6.6 years. Acute painful events, severe anemia and fever with infections were the major complications and 23 babies required hospitalization. Severe to moderate clinical presentation was found in 13.8% babies with SCD whereas, 86.2% babies had a milder presentation. Presence of ameliorating factors (α-thalassemia and Xmn 1 polymorphism) did not have a discernible effect on the clinical severity. Parents of babies with SCD were educated and counseled for home care. Distribution of mobile phones to 44 families having babies with SCD was beneficial as it allowed regular contact with patients and their families. Genetic counseling to the affected families has increased the awareness and acceptance for prenatal diagnosis and 18 couples opted for prenatal diagnosis in subsequent pregnancies. SCD is not always mild among tribal groups in India. Therefore, facilities for early diagnosis and prophylactic treatment in the tertiary care centers should be made available. The difficulties in regular follow up of the babies in remote rural areas have also been highlighted.

## Introduction

Sickle cell disease (SCD) is a major public health problem in India with a higher prevalence among the tribal and some non-tribal ethnic groups. The clinical manifestations are extremely variable ranging from a severe to mild condition. Early diagnosis and providing care is critical in SCD because of the possibility of lethal complications in early infancy in pre-symptomatic children (1).

Newborn screening (NBS) enables the identification of babies with sickle cell disease at birth or soon after, within the first few days of their life before they present with any symptoms or complications. These babies can then be regularly followed up with the provision of comprehensive care and timely management to reduce morbidity and mortality. It has been demonstrated in several countries that early diagnosis and providing care is critical in SCD as chances of lethal complications in infancy have been noted (2, 3).

It has been estimated that 50% of sickle cell heterozygous and homozygous neonates are born in Nigeria, India and Democratic Republic of Congo. Among these, India contributes to about 15% of the world's sickle cell anemia neonates. Further it has been estimated that widespread newborn screening and follow up care could save the lives of almost 10 million children by 2050 (4). Thus, newborn screening has great relevance in this country. There is no National neonatal screening program for SCD as yet in India and affected children are generally identified when they become symptomatic. However, few newborn screening programs have been initiated in some regions in the last 5–6 years (5–9).

The present study was undertaken to establish a newborn screening programme in the tribal areas of Gujarat and Madhya Pradesh to raise a cohort of babies with SCD and follow them up along with comprehensive care to understand the early morbidity and mortality of the disease.

## Materials and Methods

### Populations Group Studied

**Figure 1:**
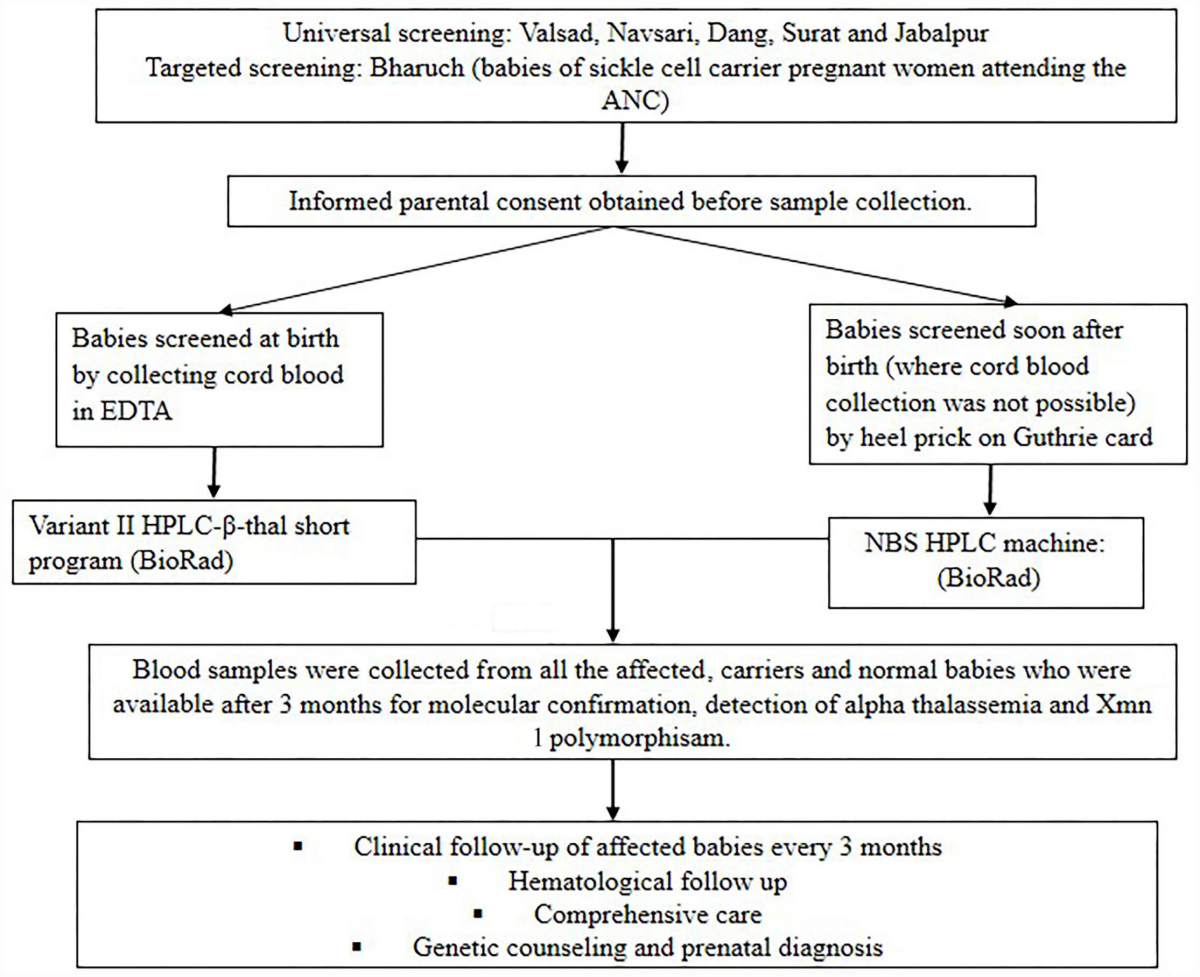
Flow chart of methodology.

Figure 1 shows the flow diagram of the methodology followed in the present study. A total of 8,916 newborns from different tribal and non-tribal groups were screened from different districts of Gujarat (*n* = 8,411) and Madhya Pradesh (*n* = 505) over six years (2010–2016). Five districts were covered in Gujarat viz Valsad, Navsari, Dang, Bharuch and Surat. Newborn screening was largely targeted to tribal women and further targeted to offspring of mothers with an AS genotype at SEWA rural hospital in Bharuch. Babies of all sequential deliveries where the parents gave their consent were screened. In Madhya Pradesh universal newborn screening was carried out mainly from Jabalpur district. Cord blood samples were collected during hospital deliveries while heel prick samples were collected by health care workers on Guthrie cards between days 1 to 7 after birth in those babies where cord blood samples were not available. The study was approved by our institutional ethics committee (IEC).

### Hematological Analysis

Complete blood count (CBC) was done on an automated cell counter (SysmexK-1,000, Sysmex Corporation, Kobe, Japan) when cord blood samples were collected. Screening for hemoglobinopathies was done by HPLC on the Variant^TM^ NBS Newborn Hemoglobin System for filter paper samples and on the Variant II HPLC machine using the β-thalassemia short programme for cord blood samples (BioRad Laboratories). Statistical analysis was done using student's *t*-test to compare the hematological findings in mild vs. moderate/severe cases.

### Molecular Analysis

DNA was extracted from the cord blood leucocytes or on follow up using the QIAamp DNA Mini Kit (QIAGEN, Germany). The sickle and β-thalassemia mutations were confirmed by Covalent Reverse Dot Blot hybridization (CRDB) (10) or by Amplification Refractory Mutation System (ARMS) (11). Detection of the common α gene deletions (-α^3.7^ and -α^4.2^) was done by multiplex polymerase chain reaction using Qiagen multiplex master mix (QIAGEN, Germany) (12). The −158(C>T) variation upstream of the Gγgene (Xmn 1 polymorphism) was detected by PCR-RFLP analysis (13).

### Clinical Follow-Up

Babies with SCD were followed-up clinically every three months as far as possible. Clinical phenotypes were defined as per the scoring system (Table 1) described earlier (14, 15). To define painful crises, mothers were specifically asked about pain and/or swelling of the fingers or toes (defined as dactyilitis) or bone pain elsewhere defined as the bone pain crises. Sepsis was defined clinically based on the integrated management of neonatal and childhood illness (IMNCI) guidelines (16).

**Table 1:** Scoring system for clinical evaluation of children with sickle cell disease.

**Complications**	**Score**				
	**0**	**1**	**2**	**3**	**5**
Hospitalizations/year	0	1	2	>3	–
Blood transfusions/year	0	1	2	>3	–
Painful events/year	0	1	2	>3	–
Dactylitis events/year	0	1	2	>3	–
Vaso-occlusive crises	No	–	–	Yes	–
Sequestration crises	No	–	–	Yes	–
Acute chest syndrome (ACS)	No	–	–	Yes	–
Sepsis	No	–	–	–	Yes
Stroke	No	–	–	–	Yes

*Classification of the patient.*

*Total score for milder patient: 0-3.*

*Total score for moderate patient: 4-7.*

*Total score for severe patient: 8-15.*

## Results

Based on the HPLC findings, in Gujarat 122 babies had Sickle cell disease whereas in Madhya Pradesh 6 babies had sickle cell disease. Of the 122 babies with SCD in Gujarat, molecular studies in 87 revealed 69 HbSS and 18 with HbS-β-thalassemia [13- Codon 15 (G → A), 5-IVS 1–5 (G → C)].

### Clinical and Hematological Follow Up

These 87 babies from Gujarat could be followed for 0.5–6.6 years. This also included 40 babies from our earlier NBS program (8) and 47 babies from the present NBS program. Clinical and hematological evaluation was done. Of the 87 sickle cell disease babies, 63 (72.4%) had one to 4 follow ups while 24 (27.6%) babies had more than 4 follow ups. Of the 87 babies 23 (26.4%) were regularly followed up at the interval of 3 months. CBC and HPLC analysis was only possible in 42 babies due to the unwillingness of parents to allow blood collection of their baby each time when they come for the clinical follow-up.

Table 2 shows the hematological parameters including HbF levels in the mild and moderate to severe cases of sickle cell anemia and sickle-β-thalassemia at the last follow up (age varied from 1 to 6.6 years). Sequential hematology was available in 42 babies 36 classed as mild, 6 moderate to severe. Significantly higher HbF levels (p < 0.05) were observed in the mild cases with sickle cell anemia compared to the moderate to severe cases. We did not find any significant difference in HbF levals in different age groups.

**Table 2:** Hematological findings during follow-up of babies with HbSS and HbS-β-thalassemia having mild and moderate to severe clinical presentation.

**Hematological parameters**	**Sickle cell anemia (n = 35)**		**Sickle-β-thalassemia (n = 7)**	
	**Mild**	**Moderate to severe**	**Mild**	**Moderate to severe**
	**(n = 31)**	**(n = 4)**	**(n = 5)**	**(n = 2)**
RBCs (x10^12^/l)	4.76 ± 0.6	3.89 ± 0.9	4.67 ± 0.4	4.50–4.58
Hb (g/dl)	8.8 ± 1.0	7.9 ± 1.7	8.7 ± 0.7	8.80–9.60
MCV (fL)	62.6 ± 8.2	72.7 ± 4.0	62.6 ± 5.9	67.0–69.6
MCH (pg)	18.7 ± 2.3	20.4 ± 0.2	18.8 ± 2.0	20.6–20.0
MCHC (g/dl)	29.8 ± 1.7	28.1 ± 1.2	30.1 ± 1.4	30.0–29.4
RDW (%)	21.6 ± 5.2	19.4 ± 4.4	21.4 ± 6.3	19.0–19.4
HbF (%)	23.5 ± 8.4^*^	8.6 ± 0.3^*^	27.5 ± 9.3	18.4–20.7

* *Statistically Significant (p < 0.05)*.

The clinical presentation of 87 babies who were followed up is summarized in Table 3. Among them, one sickle homozygous baby had Down's syndrome, one had congenital heart disease (CHD) and another presented with intellectual disability and hypotonia. Severe anemia was defined based on the Hb level (<6g/dl). Of the symptoms, dactylitis occurred in 2 (2.3%), acute chest syndrome in 3 (3.4%), bone pain crises in 19 (21.8%), febrile episodes in 26 (29.9%), sepsis in 6 (6.8%), and severe anemia in 11 (12.6%), who were treated with blood transfusion. Twenty three babies (26.4%) required hospitalization for infections, severe painful crises and blood transfusions. On an average period of hospitalization was 2–3 days. Splenomegaly was observed in 10 children (11.5%) with a spleen size of 1 to 7 cm and hepatomegaly in 3 children (3.4%) with a liver size of 1 to 4 cm. There were three deaths due to severe anemia unresponsive to hydroxyurea at age 4 years, Congenital heart disease (CHD) at age 7 years, and Down's syndrome at age 8 years. Most babies were underweight and height was retarded in three babies as compared to the age and sex matched healthy babies from the same regions (unpublished data).

**Table 3:** Clinical presentation of the babies with sickle cell disease during the follow up period (0.5–6.6 years).

**Clinical presentation**	**Sickle homozygous (HbSS) (*n* = 69)**		**Sickle-β-thalassemia (*n* = 18)**	
	**No. of babies**	**No. of episodes**	**No. of babies**	**No. of episodes**
Painful crises	12 (17.4%)	1–3	7 (38.9%)	1–2
Severe anemia (Hb <6.0 g/dl)	15 (21.8%)	1–2	3 (16.6%)	1–2
Blood transfusion	9 (13.1%)	1–5	2 (11.1%)	1–2
Fever with infection	19 (27.5%)	1–3	7 (38.9%)	1–4
Sepsis	5 (7.2%)	1	1 (5.5%)	1
Hospitalization for infection or severe painful crises	15 (21.8%)	3–6	8 (44.4%)	1–14
Dactylitis	2 (2.9%)	1	0 (0.0%)	0
ACS	2 (2.9%)	1	1 (5.5%)	1
Death	3 (4.3%)	–	0	–

**Figure 2:**
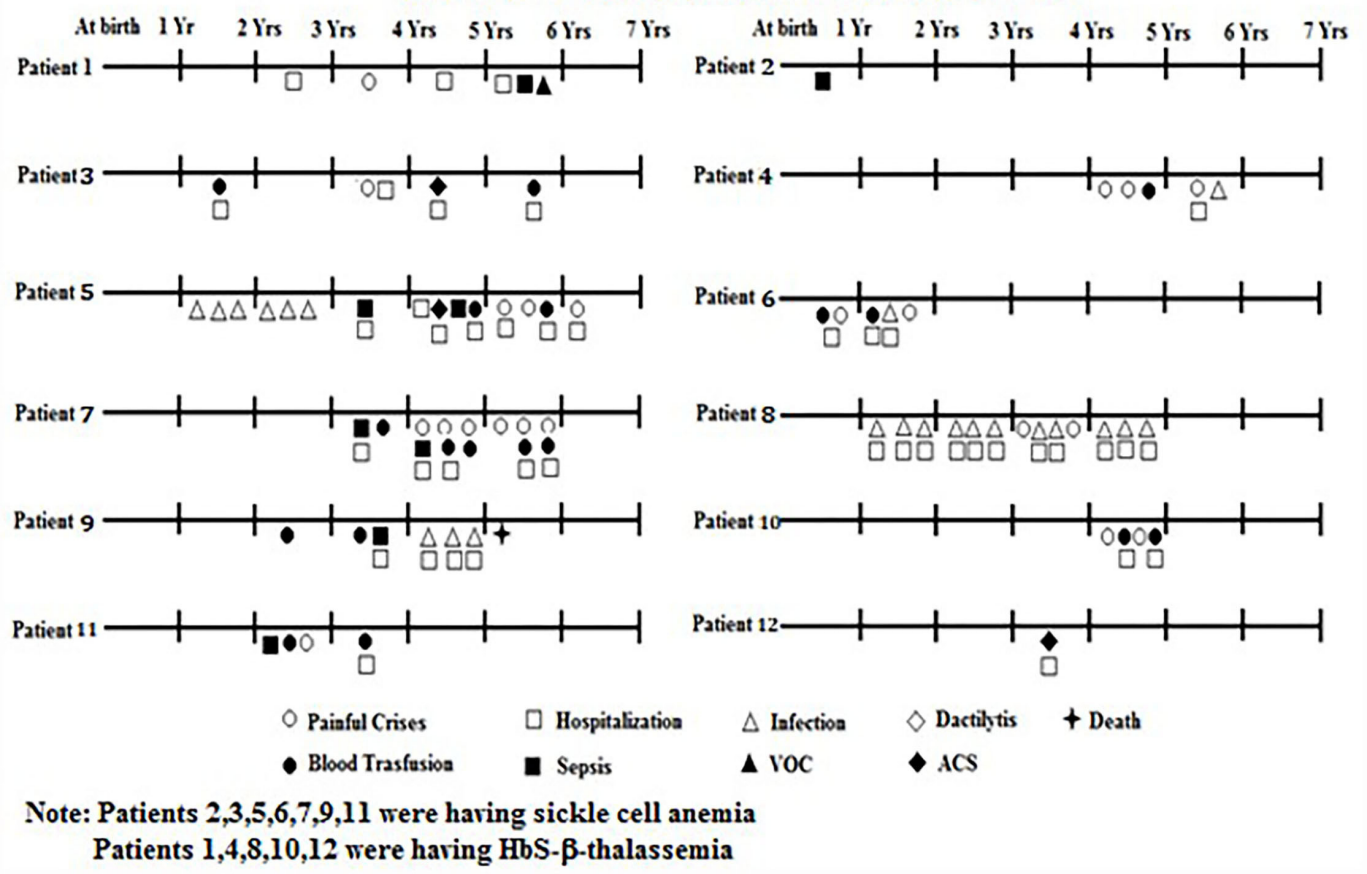
Follow Up of babies with SCD having a moderate to severe clinical presentation.

Based on the different clinical events and number of episodes mentioned in Figure 2, the babies were classified as clinically mild and moderate to severe. Of the 87 babies, 75 (86.2%) (62 HbS homozygous and 13 HbS- β-thalassemia) were clinically mild whereas remaining 12 (13.8%) (7 HbSS and 5 HbS-β-thalassemia) were clinically moderate to severe. The HbF levels were significantly higher (*p* < 0.05) among the milder babies with SCD (23.5 ± 8.4) compared to the babies with moderate to severe disease (12.3 ± 6.5). Babies with a clinically mild presentation had fewer clinical complications which included occasional painful events, anemia, mild fever with cough and cold, while in the babies with a clinically moderate to severe presentation, clinical complications such as painful events, vaso-occlusive crises, hospitalization, acute chest syndrome, severe anemia requiring blood transfusion, sepsis, and fever with infections were observed (Figure 2). The β globin gene mutation in all the moderate to severe sickle—β thalassemia babies was the severe β^0^ type of mutation Codon 15 (G**→**A).

### Comprehensive Care

Pneumococcal vaccination (23-valent polysaccharide vaccine- PNEUMOVAX_ 23) was given to all the babies after 2 years of age while folic acid supplementation of 2.5 mg was given after 1 year of age and the dose was increased to 5 mg after 2 years of age. Penicillin was not given. Analgesics were given for symptomatic pain relief. Mobile phones packed with jingles as well as all relevant information on sickle cell disease in the local language were distributed to 44 families having babies with SCD. Due to budgetary restrictions, mobile phones could not be given to all families and were restricted to those who were living in the most remote areas and those who could not visit the clinic regularly. A special questionnaire had been prepared for telephonic follow-up which was filled up during each call. A total of 33 calls were received from 14 parents to inform the center about any major life events or sickle cell related problems in the child and to inquire about the next follow-up date as well as future preventive options. Immediate advice was given by the medical officer. At the same time the genetic counselors and medical officers were in constant touch with these 44 families (Table 4). Majority of the babies had generalized weakness, loss of appetite, fatigue, fever, cold and cough. Few had severe sickle cell crises such as painful events, severe anemia requiring blood transfusions. Immediate advice given over the phone by our Medical Officer helped to reassure the parents. Immediate arrangement of blood for transfusion was also done when required for the babies with SCD staying in very remote areas.

**Table 4:** Number of phone calls received from the parents of the affected babies for different complaints.

**Complaints**	**Number of phone calls**
Generalized weakness and loss of appetite	3
Fever and pain	6
Anemia and blood transfusion	8
Cold and cough	5
Inquiry about prenatal diagnosis	2
Follow up date inquiry	9
Total	33

### Determination of the Presence of α-Thalassemia and Xmn 1 Polymorphism

Presence of α-thalassemia was determined in 83 babies. Alpha—thalassemia was found to be present in 71/73 (97.2%) babies with a mild clinical presentation (αα/-α^3.7^−8, -α^3.7^/-α^4.2^−2 and -α^3.7^/-α^3.7^−61) and in 9/10 (90.0%) babies with moderate to severe clinical presentation (αα/-α^3.7^−1, αα/-α^4.2^−1 and -α^3.7^/-α^3.7^−7). Three babies (2 mild and 1 moderate to severe) had a normal α-genotype (Table 5).

**Table 5:** α-genotype among the affected babies.

**Clinical presentation**	**αα/αα**	**-α^3.7^/αα**	**-α^4.2^/αα**	**-α^3.7^/-α^3.7^**	**-α^3.7^/-α^4.2^**	**Total**
Mild	2 (2.7%)	8 (10.9%)	0	61 (85.6%)	2 (2.7%)	73
Moderate to severe	1 (10.0%)	1 (10.0%)	1 (10.0%)	7 (70.0%)	0	10
Total	3 (3.6%)	9 (10.8%)	1 (1.2%)	68 (81.9%)	2 (2.4%)	83

The presence of the Xmn 1 polymorphism was determined in 83 (66 HbSS and 17 HbS- β-thalassemia) babies. All the babies with HbSS were homozygous (+/+) for the Xmn 1 polymorphism, a feature of the Arab Indian haplotype whereas all the babies with HbS-β-thalassemia were heterozygous for the Xmn 1 polymorphism (+/-).

### Genetic Counseling and Prenatal Diagnosis

The families of the affected children were also called and counseled for home care. Genetic counseling was also given to the parents of all 87 babies (87 couples) for prenatal diagnosis and subsequently 18 couples at- risk of having a HbSS baby where a pregnancy was confirmed opted for prenatal diagnosis. Four fetuses were found to be normal, 8 were sickle heterozygous and 6 were sickle homozygous. As all the couples had a previous child with sickle cell anemia and it is difficult to predict the clinical course of sickle cell homozygous babies, all the 6 couples opted to terminate these pregnancies.

## Discussion

India had an estimated annual birth of around 42,000 babies with SCA in 2010 mainly among the scheduled tribes and some other economically disadvantaged populations like the scheduled castes and other backward classes (1, 4). Newborn estimates are important to know the precise number of births of babies with SCD to determine the magnitude of the burden of the disease. Recently it was estimated that the number of newborns with SCA in 2020 will be the highest in Madhya Pradesh, followed by Tamil Nadu, Maharashtra, Gujarat, Odisha and Chhattisgarh (17, 18). Few newborn screening programs have been initiated in Maharashtra, Gujarat, Madhya Pradesh, Chhattisgarh, Odisha and Tripura. Most of these were pilot studies which targeted both tribal and non-tribal populations (6–9).

In the present study, defaults rates of 29% occurred in Gujarat despite our best efforts, whereas, in Madhya Pradesh no follow-up was possible due to several reasons such as unwillingness of the parents, incorrect contact details and the distance of the screening centre from their residence. This may have introduced some biases in the analysis of the clinical presentation. Of the 87 affected babies, 40 babies were identified in our earlier NBS program (8) and 47 were from the present screening program. We included the affected babies of our earlier screening program in Gujarat also as they were followed up for a longer duration. The same protocols were followed for screening and follow up in both these cohorts.

The presence of α-thalassemia was also evaluated in these babies and it was found that 63/75 (84.0%) affected babies with a mild clinical presentation and 7/12(58.3%) affected babies with a moderate to severe clinical presentations had two alpha gene deletions. Also all the babies affected with SCD had the Arab Indian haplotype. A significantly higher HbF level was observed in the milder group of HbSS cases than in the moderate to severe group of cases. Earlier study by Upadhye et.al. suggested that the influence of other genetic modifiers like certain SNPs in BCL11A and HBS1L-MYB genes that influence HbF levels were associated with a milder clinical presentation in patients with SCD from Nagpur in central India (19). However, we have not looked at these modifiers in our cohort of affected babies.

The main aim of a newborn screening programme for SCD is to provide comprehensive care to the affected babies and genetic counseling to the couples at risk to avoid further births of affected babies. In the few newborn screening programs conducted earlier it has been seen that many a times the follow–up of the affected babies was not possible because of incorrect contact details, illiteracy of parents and the distance of the health center from the residence of the family (6–9). These problems were particularly faced by us in Madhya Pradesh where the babies with SCD could not be followed up. Unfortunately, in Gujarat too during the first NBS programme there was a high rate of lost to follow up babies (30.4 %) in spite of our best efforts.

To overcome these problems we tried a new concept of providing mobile phones to the parents of the affected babies and also gave them the relevant information on SCD including the guidelines that how the parents should take care of their children at home and recognize any acute events and contact the clinic immediately. The concept of giving mobile phones was interesting especially for patients in remote areas as it allowed regular contact twice a month with patients and their families. At the same time, the parents could contact the social worker and medical officer at the center regarding any major life events or sickle cell related problems in their child. This new concept received an excellent response which helped us to follow-up all the 44 babies affected with SCD regularly. However, more data are required for sufficient evidence to establish the effect of mobile phones in these remote areas. Also, awareness and genetic counseling has led to the acceptance of prenatal diagnosis among the tribal groups. A major limitation of the study was the small number of newborn babies who could be screened in Madhya Pradesh which has the maximum burden of sickle cell disease in India and the absence of follow up of babies with SCD in this state where concentrated efforts would be required to convince the parents on the importance of newborn screening and early care. This was a limitation in Gujarat too where greater efforts would be needed to reduce the number of defaulters for a regular follow up. The target populations in both these states belong to different indigenous socially disadvantaged groups with a low literacy rate often living in rural forested areas where infrastructure is limited and they move to different areas seasonally in search of work. Reaching out to them and convincing them to allow blood collection of their infants and children repeatedly is a challenge.

Yet the main strength of the study is the feasibility of undertaking a newborn screening programme for SCD among large tribal populations in remote areas, evaluating the clinical presentation, providing them care and their acceptability for prenatal diagnosis. Thus, a major focus has to be directed toward educating them, increasing their level of awareness and offering them genetic counseling. This is the only way to move forward for successful implementation of newborn screening programmes in India with long term longitudinal follow ups of cohorts of babies with SCD.

## Conclusion

The present study demonstrates that unlike earlier belief, the disease is not always mild among the tribal groups in India. In addition, increasing awareness, comprehensive care and regular monitoring of babies with SCD could reduce the morbidity and mortality. Therefore, facilities for early diagnosis and prophylactic treatment in the tertiary care centres should be made available. This would help policy makers to develop facilities where they are most needed and implement newborn screening of SCD in the public health programmes in different states.

## Data Availability Statement

The original contributions presented in the study are included in the article/supplementary material, further inquiries can be directed to the corresponding author/s.

## Ethics Statement

The studies involving human participants were reviewed and approved by IEC of Indian Council of Medical Research (ICMR) National Institute of Immunohaematology. Written informed consent to participate in this study was provided by the participants' legal guardian/next of kin.

## Author Contributions

PT and MBM wrote the first draft of the manuscript. PT carried out all the molecular analysis. At Gujarat JP, BR, and KD were involved in HPLC analysis of newborns. VM, AM, and SD clinically evaluated the affected babies at each follow-up and provided necessary interventions. YI and SD supervised the laboratory work and follow-up of the babies. At Madhya Pradesh RS was involved in HPLC analysis of newborns and supervised the laboratory work. RBC and MBM designed the study, helped with the analysis of data and finalized the manuscript. KG provided intellectual inputs and was also involved in clinical evaluation of the affected babies. All the authors have read and approved the manuscript.

## Funding

This work was supported by the Grant Number Tribal/62/2012-ECDII dated 10/04/2013 from Indian Council of Medical Research, New Delhi.

## Conflict of Interest

The authors declare that the research was conducted in the absence of any commercial or financial relationships that could be construed as a potential conflict of interest.

## Publisher's Note

All claims expressed in this article are solely those of the authors and do not necessarily represent those of their affiliated organizations, or those of the publisher, the editors and the reviewers. Any product that may be evaluated in this article, or claim that may be made by its manufacturer, is not guaranteed or endorsed by the publisher.
